# Enhancing Plant Growth and Yield Quality Through Molybdenum Application: Trends, Future and Directions

**DOI:** 10.3390/plants15040585

**Published:** 2026-02-12

**Authors:** Kovács Béla, Eva Bodi, Szilvia Várallyay, Shaikh Ayaz Mukarram, Abdelhakam Esmaeil Mohamed Ahmed

**Affiliations:** 1 Faculty of Agricultural and Food Sciences and Environmental Management, Institute of Food Science, University of Debrecen, Böszörményi str. 138, 4032 Debrecen, Hungary; bodieva@agr.unideb.hu (E.B.); varallyay.szilvia@agr.unideb.hu (S.V.); ahmed.abdelhakam@agr.unideb.hu (A.E.M.A.); 2 Doctoral School of Food Sciences, University of Debrecen, Böszörményi str. 138, 4032 Debrecen, Hungary; 3 Faculty of Forestry, University of Khartoum, Khartoum North 13314, Sudan

**Keywords:** plant nutrients, beneficial elements, global food system, physiology of plant, crop productivity

## Abstract

This study presents a comprehensive synthesis of molybdenum (Mo) research from 1999 to 2025, analyzing 500 peer-reviewed publications from the Web of Science, with a detailed analysis of 200 documents focusing on Mo’s role in plant nutrition and growth. This integrated approach highlights the evolving scientific focus on Mo, emphasizing its biochemical functions and agronomic importance in enhancing crop production and quality. These findings underscore Mo’s critical contribution to sustainable agriculture and align with the global food security goals outlined in the United Nations Sustainable Development Goals. This review identifies key research gaps, including the need for large-scale field validation, crop- and soil-specific Mo requirements, and nutrient interaction studies under stressful conditions. Future research should prioritize multi-location trials and advanced omics techniques to optimize Mo management and improve crop resilience and yield. Limitations include reliance on English, open-access publications, and variability across studies; however, the combined bibliometric and physiological analysis offers a robust foundation for advancing Mo research in agriculture.

## 1. Introduction

Feeding billions of people on the planet requires the production of high-yield high-quality food, which is a challenge that must be addressed. Estimates show that global food production will increase by 70% by 2050, thereby increasing pressure on land and nutrients. Currently, 820 million people, mostly from developing countries, suffer from hunger and malnutrition [1,2,3]. Loss of these nutrients occurs under environmental stress and soil infertility [4,5]. The use of micronutrients, such as molybdenum (Mo), which are needed in trace amounts, is essential for promoting plant growth, nutrient absorption, and overall yield quality [6,7]. Mo is a key component of several plant enzymes, including nitrate reductase and nitrogenase, which are involved in nitrogen metabolism and biological nitrogen fixation in legumes [8,9]. These functions make Mo crucial for plant growth, chlorophyll production, and stress resistance [10,11,12], especially in crops grown on acidic or Mo-deficient soils [13,14].

Soil conditions, specifically pH, strongly affect Mo availability [15]. In acidic soils, Mo becomes less accessible to plants, often causing deficiency symptoms, such as stunted growth [16,17], leaf chlorosis, and weak nodulation in legumes [18,19]. To address these issues and enhance plant nutrient efficiency, MoAPG using soil, seed, or foliar application is commonly used in various agricultural systems [20,21,22]. Previous studies have focused on the Mo application efficiency linked to plant growth, yield, quality, and resilience under environmental stresses, such as drought and salinity [23,24]; however, no single review has combined its scientific focus and conceptual perspectives in all the mentioned aspects over time. The main aim of this review was to provide current evidence on Mo occurrence, physiological roles, uptake and translocation, deficiency and toxicity symptoms, and their interactions with macro- and micronutrient uptake across diverse plant systems. By integrating bibliometric mapping (1999–2025) with a comprehensive scientific analysis of MoAPG, this study traces the evolution of Mo research from its traditional role in deficiency correction to emerging functions in enzyme regulation, stress tolerance, and crop quality improvement.

Furthermore, this integrated approach established a new conceptual basis linking Mo metabolism to plant resilience and productivity. The review also identifies key research areas, global research capacity, and collaboration patterns; evaluates the agronomic impacts of Mo application in relation to sustainable food production; and highlights existing knowledge gaps and future research directions aligned with sustainable agriculture and the Zero Hunger goal.

The specific objectives of this study were as follows.

(A) To identify significant research areas, research capacity, country contributions, co-authorship networks, and leading publishers involved in MoAPG research using selected keywords (“molybdenum,” AND “plant growth,” AND “plant nutrition,” AND (“crop OR yield,”) AND “agriculture”)).
(B) To synthesize the current scientific findings on the physiological effects of MoAPG in different plant species and their effects on nutrient uptake, enzymatic activity, and dry biomass accumulation.
(C) To explore the agronomic influences of Mo application, such as yield quality, abiotic stress tolerance, and nitrogen fixation, to sustain the supply of foodstuffs, which is aligned with the Sustainable Development Goal of zero hunger.
(D) To identify current research gaps and outline directions for future studies on the role of Mo in sustainable crop production for agricultural practices.

### 1.1. Exclusion Strategy, Screening, and Data Extraction

**Figure 1:**
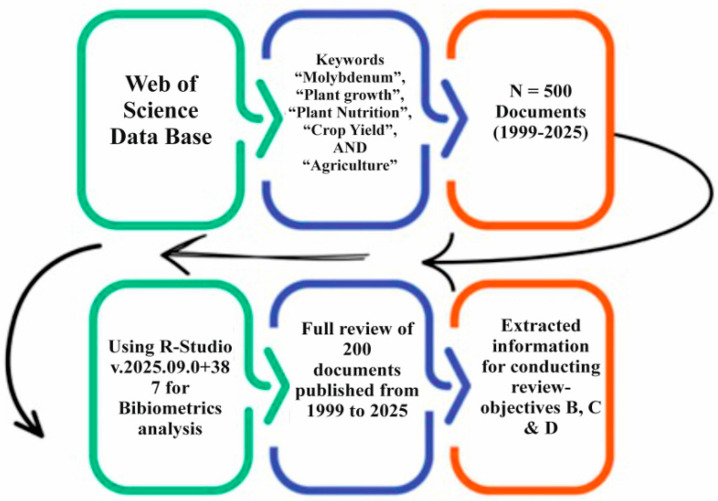
Workflow of (MoAPG) and crop production documents’ screening on the attained metadata from the Web of Science (WoS) database (1999–2025).

A literature search was conducted using the Web of Science (WoS) essential method, chosen for its high-quality indexing of peer-reviewed scientific literature on Mo [25], on 20 September 2025, using the following keywords: (“molybdenum,” AND “plant growth,” AND “plant nutrition,” AND (“crop OR yield,”) AND “agriculture”)) see (Figure 1). English-language, open-access, peer-reviewed articles, proceedings, and book chapters related to molybdenum and plant growth were included, resulting in 500 relevant publications from an initial 907 records. A bibliometric analysis was performed using RStudio (R-Studio v. 2025.09.0+387 (PBC, Boston, MA, USA) with the Bibliometric package to examine research trends, collaboration networks, and thematic evolution. Selected studies were further screened for qualitative synthesis, and numerical data presented graphically were extracted using the WebPlotDigitizer (version 5.0; Ankit Rohatgi, Austin, TX, USA). This integrated bibliometric and analytical approach enabled a comprehensive assessment of Mo research on plant growth and crop production. This integrative method allowed for the clear interpretation of scientific evidence supporting the physiological and agronomic significance of MoAPG, and was used as evidence to answer the following research questions:

- What are the main areas of research, publication volume, geographical distribution, cooperative networks, and leading publishers in the MoAPG and crop production fields?
- What are the main effects of MoAPG on plant physiological responses such as nutrient metabolism, nitrogen fixation, and enzymatic activity?
- How does MoAPG affect agronomic characteristics, including biomass accumulation, yield quality, and stress tolerance across different crop species?
- What are the recent scientific gaps, limitations, and evolving directions in MoAPG research?

### 1.2. Literature Review and Analysis

#### 1.2.1. The Bibliometric Dataset According to WoS Search on (MoAPG) and Crop Production

**Figure 2:**
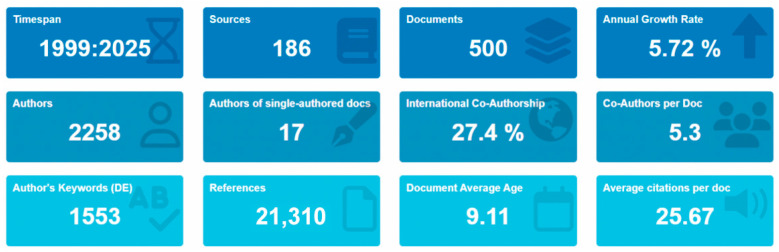
Overview of bibliometric indicators for published (MoAPG) and crop production documents from 1999 to 2025. Number of sources, documents, annual growth rate, authorship patterns, citations, international collaboration, and keyword range. MoAPG: “molybdenum, application, on plant growth”.

An overview of the bibliometric dataset is shown in Figure 2, in which 500 documents/articles referring to the total number of individual publications included in the bibliometric analysis for the period between 1999 and 2025 were obtained from 186 sources (e.g., journals, conferences, proceedings, and books). Annual scientific production exhibits an outstanding annual growth rate of 5.72%, representing cumulative research in the MoAPG field over the past two decades. The publications were authored by 2258 authors, with a relatively low number of single-author publications (17 authors), suggesting strong international co-authorship and collaboration. The average document had 5.3 co-authors, and international coauthorship was intricated in 27.4% of the documents examined, displaying the global nature of research in (MoAPG) and crop production.

#### 1.2.2. Years of Publications and Publishing Progress of MoAPG Documents

**Figure 3:**
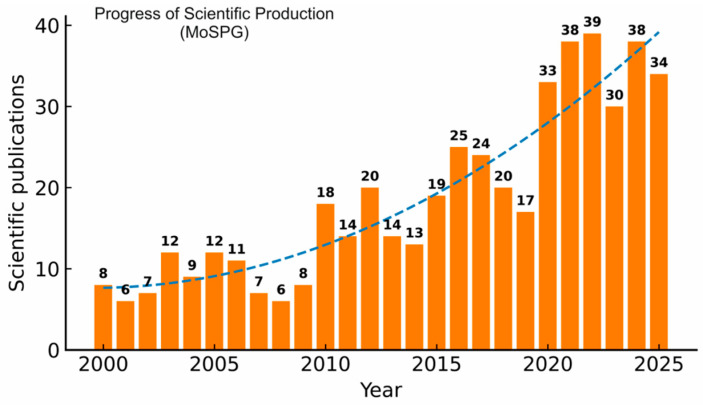
Annual scientific production related to molybdenum application on plant growth (MoAPG) and crop production within the field of plant nutrition from 1999 to 2025. Data were extracted from the Web of Science Core Collection. Bars represent the number of publications per year, while the dashed line indicates the fitted temporal growth trend. The coefficient of determination (R^2^ = 0.8429) reflects the strength of the publication growth over the study period.

Figure 3 shows the temporal distribution of scientific publications on the role of Mo in enhancing plant growth, nutrient metabolism, and yield. The number of publications remained relatively low and stable between 1999 and 2008, with a maximum of ten documents per year. A notable upward trend was observed with a slight increase in publication production after 2020 (*n* = 20). The highest output was recorded in 2022, with 39 publications, and a notable drop in 2019 (*n* = 17 publications). However, the R^2^ value of 0.8345 shows exponential progress, but the number of publications is still low, highlighting the need for growing academic and practical interest in the field of molybdenum application, and its role in plant physiology as a timely and appropriate research area is expected to contribute to agricultural technologies, international food security concerns, and research programs advancing sustainable growth and productivity. This research aligns with the Sustainable Development Goal of zero hunger to support the world’s population, which is expected to exceed 9 billion by 2050, and food production is expected to increase by more than 85% [26,27,28].

#### 1.2.3. The Most Corresponding Authors and Top Cited Countries of the MSA Documents

**Figure 4:**
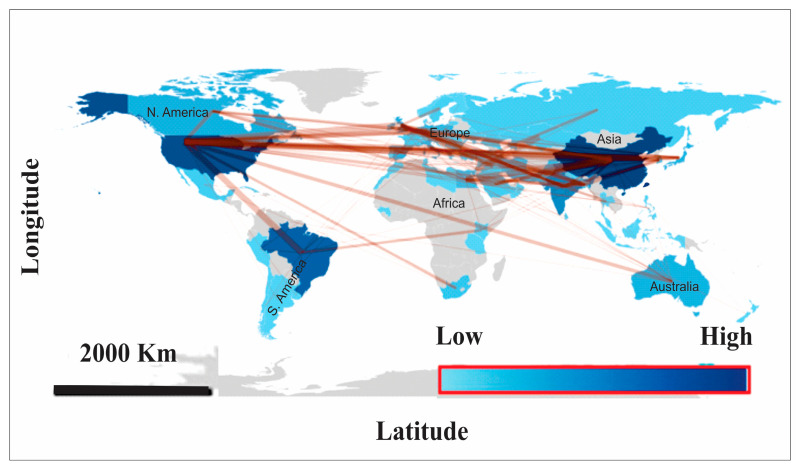
The map demonstrates international collaboration patterns based on corresponding authors’ countries in publications related to (MoAPG) and crop production (1999–2025). Dark color indicates higher productivity. Lines characterize cooperative links between countries, and their thickness relates to the strength of co-authorship.

Collaborative partnerships and mapping research trends have become scientometric analyses [29]. Therefore, the analysis of the corresponding authors’ countries in (Table 1 and Figure 4) reveals a clear geographic distribution of MoAPG research output and collaborative relationships. China had the highest output (80 articles; 16%), mainly through a large number of single-country publications (SCPs = 56), showing a strong national research base of MoAPG, which is aligned with [30] and multiple international collaborations (MCPs = 24; 30%). Brazil followed with 67 articles (13.4%), with most being produced domestically (85% SCP), reflecting a research culture focused inward, with limited international engagement (MCP = 14.9%). The United States (53 articles; 10.6%) displayed a more stable outline with a high number of multicountry collaborations (MCPs = 28.3%), indicating dynamic global networking. India has an important influence (39 articles; 7.8%) but shows very low international teamwork (MCP = 5.1%), representing domestic authorship.

Smaller but substantial sponsor countries, such as Canada (35% MCP), Australia (26.3% MCP), and Italy (40% MCP), illustrate practical-to-high international collaboration despite lower overall production. Remarkably, the United Kingdom (66.7% MCP) and Israel (66.7% MCP) stand out, with the highest collaboration rates, showing their dependence on cross-border networks to boost research importance. In addition, Korea (80% MCP) established the strongest international orientation, although there were only a few publications (five articles). Countries such as South Africa, the Czech Republic, and Mexico are fully associated through national collaboration (100% SCP), suggesting their limited incorporation into international research networks. The varied outlines across European countries (Poland, Spain, France, and Germany) specify a mix of local strengths and moderate collaboration.

The use of elemental nutrition characteristics in the role of Mo in crop production is needed to increase the dataset and scientific production in this area through national and international collaborations [31,32].

The citation analysis presented in Table 1 shows substantial differences between the countries in terms of their research contributions and views. Japan ranked highest for total citations (TCs = 2059), with the highest average cited article of 158.4, highlighting that although its output volume is ordinary, published works are of outstanding influence and are widely referenced. China, despite being the most prolific in terms of article count, accumulates 1877 citations with a much lower average per article (23.5), indicating high productivity but rational citation impact. Brazil also has many inventions (1100 citations in total) but a low average influence (16.4), highlighting the trend of quantity over quality in citation impact.

The United States has both high total citations (1778) and a strong average impact (33.5), showing a balance between output and influence. In particular, the United Kingdom appears to be a major player in research prominence, with 1193 citations and a high average citation rate (99.4), underlining the global stimulus of fewer but high-quality publications. Midlevel European contributors such as Germany (45.3 average), France (28.2), Portugal (37.3), and Belgium (36.0) have a consistent citation influence despite lower total counts.

Countries with lower productivity but unexpected average effects, such as the Philippines (105 averages), Israel (38.1), and Portugal (37.3), are significant for their ability to have highly dominant studies, even with limited article numbers. In contrast, India (5.7), Australia (11.5), and Poland (12.4) had low citation averages, representing limited international insight into their research, irrespective of moderate output (Table 2).

Generally, the data demonstrate two different outlines: (i) countries such as China, Brazil, and India lead in volume with citation visibility, and (ii) countries such as Japan, the UK, Israel, and the Philippines realize extremely high influence given their production. These different features highlight the meaning of research quality, international collaboration, and global networking in the principle of citation impact, rather than publication volume alone.

#### 1.2.4. All Frequent Keywords Occurrence and Thematic Coupling Analysis Significance of (MoAPG) and Crop Production from 1999 to 2025

##### Frequent Keyword Occurrences

**Figure 5:**
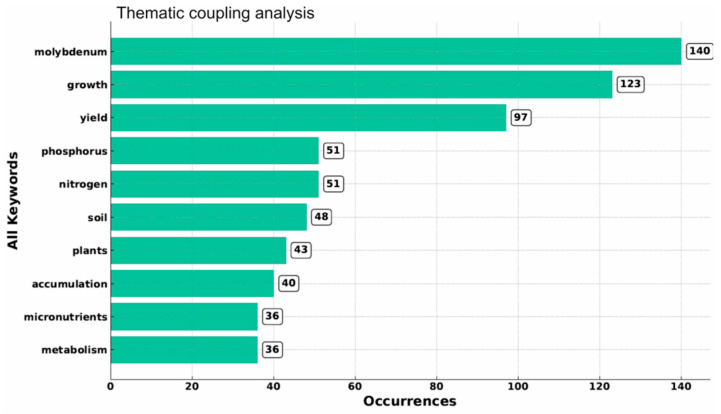
Thematic coupling analysis showing the frequency and co-occurrence strength of the most recurrent keywords in molybdenum application on plant growth (MoAPG) and crop production research from 1999 to 2025. Data were extracted from the Web of Science Core Collection. Bars represent the total number of keyword occurrences across the analyzed documents, highlighting dominant research themes such as molybdenum, plant growth, yield, soil, and nutrient interactions. This analysis illustrates the central thematic structure of the field and supports the identification of major physiological and agronomic research domains.

Keyword frequency analysis, as shown in Figure 5, provides a thorough understanding of the thematic structure and research implications of this field. “Molybdenum” occurs as the most frequent term (140 occurrences), confirming its central role as the vital element of investigation across several studies. Thoroughly related to this, “growth” (123) and “yield” (97) state that much of the research has highlighted the functional role of Mo in enhancing plant growth and crop production.

Nutrient-associated standards, such as “nitrogen” (51), “phosphorus” (51), and “micronutrients” (36), imitate the strong suggestion of Mo research with nutrient relations and soil fertility management, regularly within the broader framework of plant nutrition. The difference between “soil” (48) and “plants” (45) underlines the standing of Mo distribution, availability, and bioaccumulation in agroecosystems, which are linked to agronomic perceptions.

From a functional perspective, the occurrence of “accumulation” (40) and “metabolism” (36) reveals a growing research focus on the physiological and biochemical areas over which molybdenum influences plant function, nutrient incorporation, and metabolic directions.

In conclusion, the frequently mentioned words recommend a research area focused on the dual themes of Mo’s agronomic role in crop growth and yield, and its biological role in nutrient metabolism and accumulation within soil to plant systems. This indicates that while efficacy results remain the driving fear, physiological mechanisms are gaining cumulative attention, reflecting a steady shift toward integrative methods in molybdenum research and aligning with what was stated by Jyoti and Roy (2024) and Rana et al. (2025) [33,34].

##### Thematic Coupling Analysis

**Figure 6:**
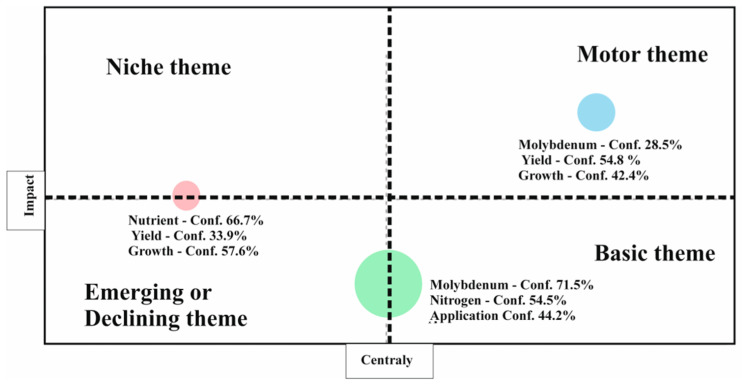
Clustering by coupling for frequent keyword occurrences of MoAPG and crop production research based on vertical axis highlighting the (Impact), and horizontal axis showing the centrality, four quadrats were shown to calcify themes as niche theme upper left, motor theme upper right, emerging or declining theme lower left and basic theme lower right. Colored circles indicate thematic clusters resulted form the analysis identified by their main keywords related with the percentage of confidence value. The blue cluster appears with high certainty and impact in the field.

In addition to frequent keyword occurrences (Figure 6), clustering using coupling analysis revealed four main thematic groups with different scientific roles. In the upper-right Q, a collection composed of molybdenum 28.5%, growth 42.4%, and 54.8% yield occurred as a motor theme, reflecting its strong centrality and impact. This shows that the association between molybdenum and plant efficiency is both well established and central to the field, which is a driving research direction. In contrast, the lower-right Q groups “molybdenum 71.5%, nitrogen 54.5%, and application 44.2%”, which characterize basic and cross themes. Although less novel, these topics form the scientific basis of molybdenum research, mostly through their roles in nitrogen metabolism and fertilizer applications, by connecting various areas of plant science. The upper-left quadrant, covering growth 57.6%, yield 33.9%, and nutrient 66.7%, signifies highly advanced connections, but in isolated niche themes. These keywords are advanced but remain less consistent with broader molybdenum-centered research, signifying opportunities for a stronger conceptual connection. In particular, the lower-left quadrant, which obviously replicates evolving or deteriorating themes, remains unoccupied, indicating that weakly established or marginal areas have not yet been projected in this field. Coupling analysis highlights the growth and criticality of molybdenum-attentive research while directing gaps between nutrient-exact yield studies and the broader context of plant physiology.

#### 1.2.5. The Temporal Evolution of Keywords and Trend Topics of the MoAPG Research

**Figure 7:**
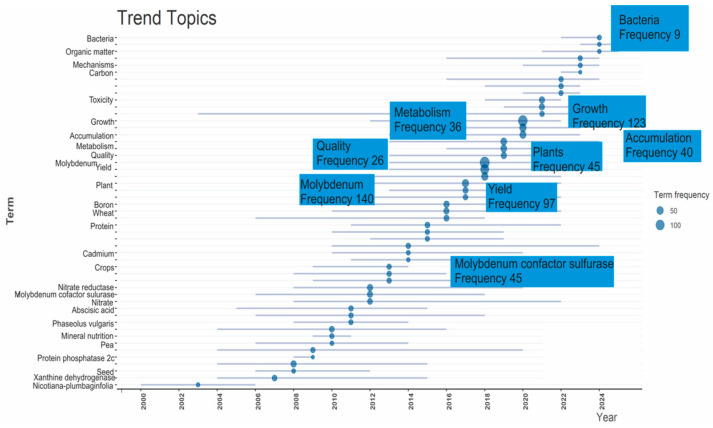
The temporal evolution of keywords and trend topics of MoAPG and crop production from 1999 to 2025, showing a dynamic shift in research attention on Mo and related topics. The occurrence and frequency of blue-highlighted keywords underscore the scientific interest of MoAPG with crop productivity.

The temporal evolution of keywords showed a dynamic shift in research attention on Mo and related topics, moving from early molecular and enzymatic studies to more recent agronomic applications, as shown in (Figure 7) in the early 2000s (2000–2009), and was dominated by model plants and enzymatic systems, such as Nicotiana plumbaginifolia (2000–2006), xanthine dehydrogenase (2004–2015), and aldehyde oxidase (2004–2015). This reflects the foundational work on molybdenum-dependent enzymes and cofactor biochemistry, which is often conducted in controlled laboratory settings.

From 2008 to 2015, emphasis was placed on hormonal adjustment and nutrient exchange using terms such as abscisic acid and its biosynthesis (2005–2018), protein phosphatase (2008–2009), and molybdenum cofactor sulfurase (2006–2018). Crop-oriented research has been conducted in pea (2006–2014), *Phaseolus vulgaris* (2008–2014), and seed studies (2006–2012). This phase represents the transition from molecular mechanisms to practical agricultural purposes.

Between 2010 and 2020, the literature slowly emphasized macronutrients and crop performance, with increasing interest in nitrate reductase (2008–2020), boron (2011–2020), and wheat (2010–2022). Since 2016, a pronounced trend toward field-scale and applied research has emerged. Overall, the trend progression displays a clear shift from the molecular mechanisms of molybdenum-dependent enzymes to their role in crop production, soil fertility, and sustainable agriculture under stress orders, with the dominant topics by frequency showing that molybdenum (frequency 140) reveals the most prominent research theme within the analyzed data. Its occurrence underscores the scientific interest in understanding its associated role in plant growth and productivity.

#### 1.2.6. The Factorial Analysis in MoAPG and Crop Production Research (1999–2025)

**Figure 8:**
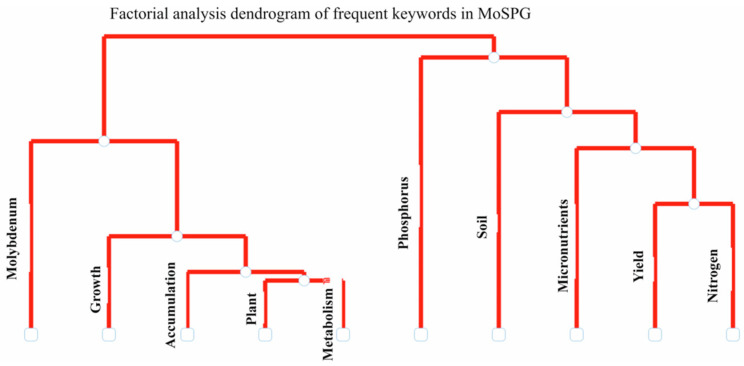
Factorial analysis dendrogram of frequent keywords in MoAPG and crop production research. Two major thematic clusters are visible: (i) a plant physiology dimension linking molybdenum with growth, accumulation, and metabolism, and (ii) an agronomic–soil dimension connecting phosphorus, soil, micronutrients, nitrogen, and yield.

Factorial analysis revealed two major thematic branches in MoAPG-related research, as shown in (Figure 8). The first branch characterizes the plant–physiological emphasis, where keywords such as “molybdenum, growth, accumulation, plants, and metabolism” cluster together, showing the biological role of molybdenum in plant growth, its accumulation in tissues, and its influence on metabolic progression. This grouping suggests that most previous studies examined the mechanistic pathways linking nutrient uptake with plant growth and biochemical characteristics. The second branch indicates the soil–nutrient–output focus, with phosphorus, soil, micronutrients, yield, and nitrogen. This cluster replicates an agronomic perspective, emphasizing the association between Mo and soil fertility and other nutrients, its relationship with nitrogen metabolism, and its effect on crop yield. The connection with phosphorus adds points to collective studies on nutrient exchanges within soil–plant systems. Factorial analysis shows that molybdenum research is designed around two reverse scopes: its mechanistic role in plant physiology and metabolism and its agronomic consequences through soil nutrients that influence plant yield.

#### 1.2.7. Multiple Correspondence Analysis (MCA) of Selected Keywords in MoAPG Research

**Figure 9:**
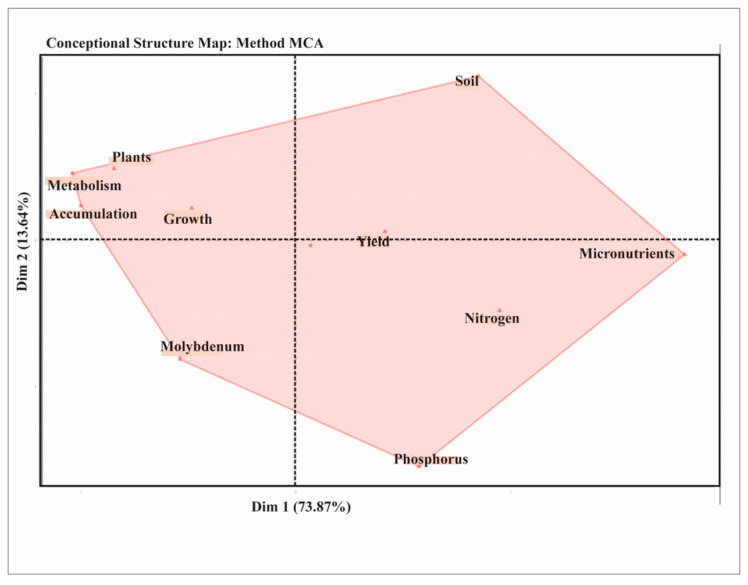
Conceptual structure analysis of selected keywords in MoAPG and crop production research through multiple correspondence analysis (MCA), showing the multiple correspondence analysis (MCA) which dim1 explained 73.87%, and dim2 13.64% of the total variance of the data offers a graphic illustration of how the MoAPG research themes were structured around the selected keywords, indicating two dominant knowledge domains such as agronomic and physiological progressions.

Figure 9 shows the conceptual structure map of multiple correspondence analysis (MCA), in which dim1 explained 73.87% and dim2 13.64% of the total variance of the data, offering a graphic illustration of how the MoAPG research themes were structured around the selected keywords. The analysis exposes two dominant knowledge domains: one attentive to broad agronomic and physiological progressions, such as soil, plants, growth, metabolism, and accumulation, and the other is a more focused area centered on nutrients, including micronutrients, phosphorus, nitrogen, and molybdenum. The essential positions of yield and growth demonstrate their role in joining concepts that connect nutrient-specific studies with wider agronomic themes. Terms located on the side line, such as molybdenum and phosphorus, signify more particular areas of research, whereas those situated at the top of the map, such as soil and plants, specify stronger incorporation within the field. The map shows that, while molybdenum-related studies form a diverse and attentive niche, they remain closely linked to plant nutrition, crop yield, agricultural productivity, and crop quality.

#### 1.2.8. All Keyword and Co-Occurrence Network in MoAPG Research

**Figure 10:**
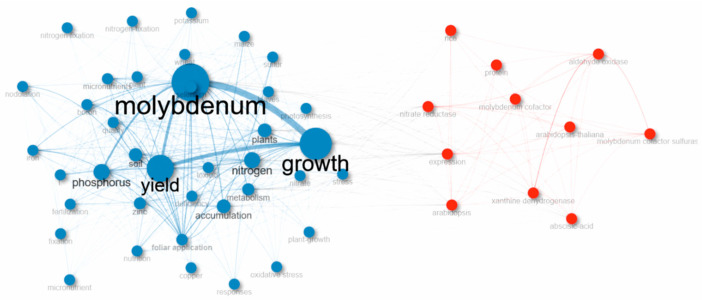
Co-occurrence network, all keywords in MoAPG and crop production research. Molebdenum, growth and yield are the most important keywords.

The keyword co-occurrence map is shown in Figure 10, which indicates two different research domains in MoAPG. The blue cluster was dominated by terms such as molybdenum, growth, plant, nitrogen, and metabolism, demonstrating that studies have focused on the biological and physiological functions of Mo, mainly its role in nutrient uptake, nitrogen metabolism, and plant development at the cellular level. The red cluster centers on keywords such as molybdenum cofactors and nitrate reductase, showing research on field-level practices in which Mo is applied via soil treatments and fertilizers to improve crop efficiency. Linking keywords, such as soil and foliar applications, relates to these two domains, viewing how physiological insights connect with agronomic observations. The most frequent and central keywords, molybdenum, plant, growth, and application, underscored the essential emphasis on the role of elements in plant growth and yield development. Whereas the blue cluster shows compressed networks, representing strong interrelationships among plant physiological concepts, the red cluster is more widespread, capturing diverse experimental and field applications. In conclusion, the visualization revealed a continuum between basic plant science and applied agriculture, highlighting Mo as the key element linking fundamental physiological processes with practical strategies for improving crop performance and yield quality.

#### 1.2.9. Percentage of Publications Addressing Sustainable Development Goals (SDGs)

**Figure 11:**
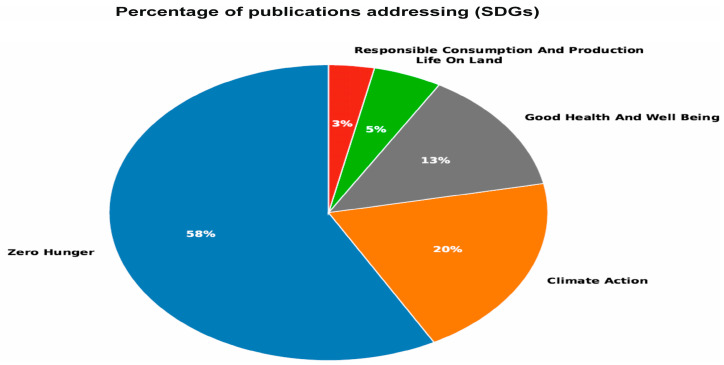
Percentage of publications addressing sustainable development goals (SDGs). The highest percentages are likely linked to SDG 2 58%), highlighting zero hunger, and SDG 12, which focuses on responsible consumption and production. SDG 13 (Climate Action) and SDG 15 (Life on Land).

Figure 11 shows the percentage of publications related to MSCP aligned with specific United Nations Sustainable Development Goals (SDGs). The highest percentages are likely to be linked to SDG 2 (58%), highlighting zero hunger, and SDG 12, which focuses on responsible consumption and production. SDG 13 (Climate Action), and SDG 15 (Life on Land). This shows that the body of research contributes meaningfully to the goals related to food security, sustainable agriculture, and environmental protection. Alignment with the SDGs highlights the practical implications of MoAPG research in addressing global challenges such as diet and nutrient effectiveness, sustainable farming practices, and ecosystem resilience.

#### 1.2.10. Leading Academic Publishers in MoAPG Research

**Figure 12:**
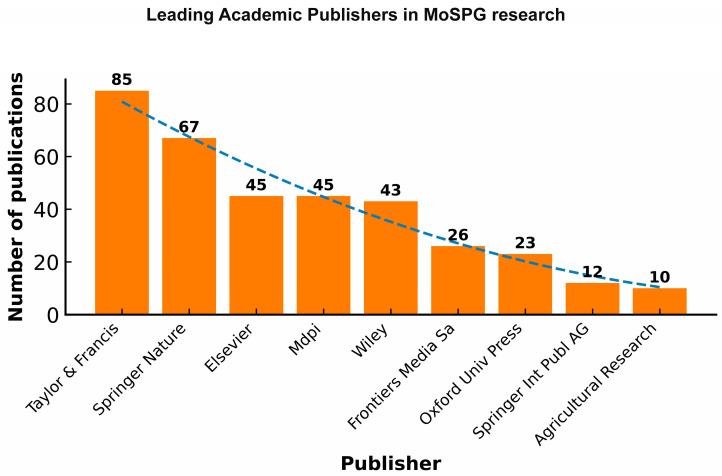
Leading academic publishers in MoAPG research. R^2^ = 0.9440. Taylor & Francis leads with the highest production of 85 publications, followed by Springer Nature with 67 publications, making them the two most dominant publishers in this MoAPG research area.

Below, Figure 12. shows the analysis of publications by publishers, demonstrating that Taylor and Francis lead with the highest production of 85 publications, followed by Springer Nature with 67 publications, making them the two most dominant publishers in this MopSPG research area. Elsevier and MDPI provided an equivalent input of 45 publications each, whereas Wiley followed with 43 publications. Other publishers such as Frontiers Media SA (26), Oxford University Press (23), Springer International Publishing AG (12), and Agricultural Research (10) subsidize at reasonably low levels. The deteriorating trend line (R^2^ = 0.9440) shows a strong fit, highlighting a concentration of research output among a few major publishers, while contributions dropped significantly. This outline replicates the central role of leading academic publishers in distributing research, and the limited but outstanding connection between smaller or specific outlets.

### 1.3. Narrative Review Overview

The bibliometric results presented in the previous sections provide a quantitative foundation for the narrative synthesis that follows. The identified thematic clusters, temporal trends, and keyword networks highlight the dominant physiological, agronomic, and soil–nutrient dimensions of molybdenum application on plant growth (MoAPG). These findings guided the structure of the following section through a critical narrative review highlighting the physiological mechanisms and agronomic outcomes, ensuring a coherent and comprehensive understanding of MoAPG research.

#### 1.3.1. Molybdenum Occurrence and Its Role in Plants

The occurrence of Mo in plants is important for mapping its role in plant physiology [35]. Mo was discovered to be important for the growth of the bacteria Azotobacter chroococcum and Aspergillus niger and fixation of atmospheric nitrogen in biological aspects [36,37]. Mo is required for plant growth [38,39,40]. Mo is essential in higher plants [7,41]. Small amounts of Mo have been shown to promote the development of medium-grown barley (*Hordeum vulgare* L.), lettuce (*Lactuca sativa* L.), and spinach [13,42,43].

Molybdenum is an essential element, and its deficiency causes symptoms in plants [9,44]. Mo deficiency symptoms that appear in plants can only be eliminated by the addition of Mo [41]. This implies that Mo cannot be replaced by any other micro-element. Furthermore, it was found that, although only a small amount of Mo was needed to eliminate deficiency symptoms in the affected plants, applying much higher concentrations increased the accumulated Mo in the shoots and roots of plants. For example, maize seedlings did not interfere with plant development, and toxic symptoms were caused only by a Mo concentration of >10 mg dm^−3^ [45].

#### 1.3.2. The Required Molybdenum for Physiological Functions and Enzyme Processes

Mo is a vital element for the growth of all plants. However, most plants require only a comparatively small amount of Mo for the smooth functioning of the plant organism. Within cereals, the molybdenum content of cereal seeds does not vary greatly, nor do their species or field growing conditions meaningfully affect their value, which is usually approximately 0.5 mg kg^−1^ (Table 3). One of the lowest values was the Mo content of spring barley grown on non-limed soil in Poland, which ranged from 0.230 to 0.340 mg kg^−1^ [46], but 0.72 mg kg^−1^ was detected in those from Russia [47]. Among the cereal crops shown in Table 2, wheat had the highest Mo content (2.4 mg kg^−1^), revealing that plant organisms require less Mo for physiological functions and enzymatic processes than other microelements (Table 3) [8,47,48].

However, the Mo content of the different plant species varied significantly, as shown in Table 4. Leguminous plants’ Mo content usually significantly exceeds that of other plants’ Mo content [49,50]. The average Mo content found in the dry matter of stover cowpeas ranged from 108 to 132 mg kg^−1,^ and that of grains was 71.6 to 109 mg kg^−1^ [51]. As tubercle bacteria on the roots of leguminous plants are responsible for nitrogen fixation, plants with leguminous flowers require high concentrations of molybdenum [52,53]. The nitrogenase enzyme plays the most important role in nitrogen binding, and the nitrogenase enzyme consists of two proteins, one containing iron and Mo, while the other contains only iron [54,55]. Rhizobium requires a relatively high amount of Mo, and pea root tubers, for example, contain approximately ten times more Mo than their aboveground parts [8,56]. Based on this, it can be stated that Mo is found in large quantities not in higher plants, but in tubercle bacteria symbiotic with it.

Mo is essential for these bacteria to bind nitrogen [57]. It is also important to mention that the amount of Mo required by plants depends on the amount of nitrogen the plant absorbs [58]. The form of nitrogen used to supply nitrogen to plants greatly influences the amount of Mo required. Most Mo is required when the nitrate form is available to supply nitrogen to plants [44]. Because Mo plays a central role in nitrate reduction, plants with nitrogen fertilization in the nitrate form require more Mo [59,60].

#### 1.3.3. Factors Affecting the Concentration of Molybdenum in Plants

In addition, the amount of Mo in plants varies with age [61]. The Mo content of plant species is inversely related to the age of the plants; younger plants had a higher Mo content, whereas the same plant contained a lower concentration when it was older [41] (Table 5).

The concentration of Mo in plants is significantly correlated with soil properties including pH, redox potential, organic matter content, and texture [30]. Mo becomes more bioavailable under neutral to alkaline conditions; however, its availability declines in acidic soils because of the formation of less soluble forms of molybdate [62]. This behavior contrasts with that of many other micronutrients, which are often more available at lower pH values [63,64]. For instance, ref. [65] reported that molybdate adsorption increases under low-pH conditions, reducing its mobility and plant uptake. Liming acidic soils significantly improves molybdenum uptake by pasture species, such as white clover (*Trifolium repens*), to improve the quality of the final products [66]. Additionally, crop species such as rapeseed (*Brassica napus*), wheat (*Triticum aestivum*), soybean (*Glycine max*), and maize (*Zea mays*) have shown variable Mo uptake efficiencies depending on the soil pH and management practices [67,68,69].

In addition to pH, soil organic matter and aeration status also play vital roles in Mo availability. References [70,71] demonstrated that molybdate binds weakly to organic matter, indicating that Mo remains relatively mobile in soils rich in dissolved organic carbon, particularly in well-aerated environments. Soil conditions determine the extent to which plants accumulate Mo, even when total soil Mo levels remain constant [72].

According to Adhikari [41,42,73], alfalfa stores large amounts of Mo in root tubers, but the Mo content in the stems and leaves is relatively low. However, the concentrations of Mo in the stems and leaves of red and Swedish clover exceeded the values measured in the root tubercles (4.6–22.7 μg kg^1^).

#### 1.3.4. Molybdenum Absorption and Translocation in Plants

Molybdenum absorption and translocation of soybeans in the initial phase of plant development, the molybdenum content increased equally in all plant parts, but was highest in the root tubers [74,75]. Initially, the molybdenum necessary for the proper functioning of root tubers came mainly from the seeds and only a small part from the soil. Moreover, the Mo content in the seeds was first translocated into the shoots and roots and then into the root tubercles. However, during the phenophase of pod formation, Mo concentrations in the roots and soybean sprouts decreased steadily until mid-June, began to increase rapidly until mid-July, and then decreased again. It was also shown that as the Mo content of soybean roots and pods decreased, the Mo concentration in pea seeds steadily increased, suggesting that some Mo was translocated from these organs into the seed crop. In Mo deficiency, root tuber formation in legumes can often be omitted, resulting in extremely low concentrations of Mo in roots [41].

The roots of plants take up molybdenum in the form of molybdate anions (MoO_4_^2−^), which is a metabolically controlled process in plant tissues [76,77]. The translocation of Mo within aquatic plants is moderate, probably owing to molybdenum-forming Mo-S complexes with sulfur-containing amino acids. Because molybdenate (MoO_4_^2−^) and sulfate (SO_4_^2−^) are chemically similar, sulfur compounds inhibit the biological incorporation of molybdenum into plants [78,79]. The distribution of Mo within plants varies depending on the plant species, but the highest concentrations of Mo are usually found in the seeds and root tubers of nitrogen-fixing plants [9,32]. Green peas grown in a nutrient solution without Mo had more root tubers, but their raw weight was lower than that of plants supplemented with molybdenum [80,81].

#### 1.3.5. The Physiological Significance of Molybdenum in Plants

In biological systems, Mo alone cannot be catalytically active unless it forms a complex bond with a specific cofactor [82,83]. In addition to bacterial nitrogenase, molybdenum is bound to pterin (molybdopterin), which is a cofactor for a series of enzymes (MOCO) [84,85]. In prokaryotes and eukaryotes, including parasites, most yeasts contain Mo as a catalytic factor for several enzymes [86,87]. All enzymes that catalyze redox reactions in plant metabolism are directly associated with changes in the oxidation state of Mo [88,89].

The physiological significance of Mo is an important component of enzymes involved in nitrogen metabolism [90,91,92]. Mo is a metal component of the nitrogenase enzyme that effectively participates in the fixation of atmospheric N. However, as a component of nitrate reductase, it plays a key role in nitrate reduction because Mo is essential for the activity of nitrate reductase [93,94]. In the absence of Mo, nitrate reduction slows, resulting in nitrate accumulation [41,95]. The importance of Mo in xanthine dehydrogenase, which plays an important role in purine metabolism [96,97], aldehyde oxidase, which is involved in the final step in the biosynthesis of indoleacetic acid and abscisic acid [57,98], and sulfite oxidase, an enzyme that oxidizes sulfite to sulfate in the metabolism of sulfur-containing amino acids [97,99]. Additionally, molybdenum inhibits the activities of deoxyribonucleic acid (DNA) [100,101] and ribonucleic acid (RNA) [102,103].

**Figure 13:**
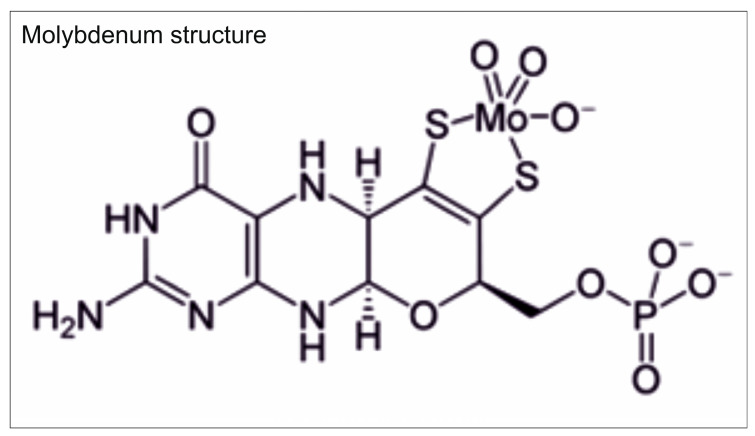
Molybdenum structure is involved in redox reactions of various enzymes.

Among these enzymes, Mo (except for nitrogenase) is present as a complex compound, the Mo cofactor (Moco, molybdopterin) [104,105]. The Mo cofactor is a heterocyclic compound in which the pterin skeleton is attached via the Mo side chain and a terminal phosphate ester is attached to the pterin skeleton [106]. In the Mo cofactor, Mo binds to the heterocyclic ring via the sulfur atoms [107,108]. The Mo structure, as shown in Figure 13, is involved in the redox reactions of various enzymes, during which the oxidation number varies from +4 to +6 [76,84].

#### 1.3.6. Impacts of Molybdenum Supply and Optimal Concentrations on Different Plants and Crops

Molybdenum is a nutrient required by plants in small amounts [58,109]. Sufficient amounts of Mo in plants vary widely according to the plant part, and most plants have the necessary levels of Mo in the range of approximately 0.2–2.0 mg kg^−1^ (on a dry matter basis) (Table 6). However, the difference between the critical deficiency and toxicity levels can be as high as four orders of magnitude (approximately 0.1 and 1000 mg kg^−1^ dry matter) [13,110].

#### 1.3.7. Molybdenum Deficiency and Symptoms in Plants

Mo deficiency usually occurs in acidic soils where Mo is less accessible to plants [117,118]. Occasionally, Mo deficiency can occur in weakly acidic or neutral soils if the original Mo content in the soil is extremely low [119,120]. The amount of Mo that can be absorbed can be increased by liming [44,121]. Liming increases yield. However, if molybdenum is added to the soil in addition to lime, an even greater effect can be achieved [122,123,124]. It should be noted that forest soils are characterized by similar conditions (molybdenum supply) compared to the above situation of agricultural soils, and the improvement of molybdenum supply in them can also be carried out in the manner detailed above [70,125,126].

A lack of micronutrients (e.g., Fe and Zn) usually appears in young leaves of plants. Most Mo is found in the xyandlauisa bealem and phloem, and it translocates relatively easily within plants. An exception to the above rule is the appearance of Mo deficiency, which does not appear in young leaves of plants but in the whole plant [9,127,128]. Symptoms of Mo deficiency are related to nitrogen metabolism; in the absence of Mo, nitrate reductase activity decreases, resulting in less or no nitrogen incorporation into proteins [13,129,130]. The characteristic symptoms of Mo deficiency are grayish-green or yellowish leaf color, chlorosis between leaf veins, chlorotic lesions starting from leaf edges, and brownish necroses that may form in chlorotic tissues [131,132]. Molybdenum deficiency in legumes is also manifested in these symptoms, as the lack of this microelement prevents nitrogen fixation by tubercle bacteria in the roots of plants [18,133].

Leguminous and cruciferous plant species have high Mo requirements because of their important roles in plant metabolic development, such as nitrate assimilation and biological nitrogen fixation. In legumes, Mo is a vital component of the nitrogenase enzyme complex in root nodules, facilitating the conversion of atmospheric nitrogen to ammonia, which is a process central to their productivity. Consequently, Mo deficiency in legumes such as lucerne (alfalfa), soybean, pea, chickpea, and clover leads to poor nodulation, chlorosis, and significantly reduced nitrogen fixation [8,56]. Similarly, cruciferous crops such as cauliflower, cabbage, and turnip exhibit severe deficiency symptoms, such as “whiptail” in cauliflower, under Mo-deficient conditions, especially in acidic soils where Mo availability is naturally low [134]. While cereals such as barley can often grow normally in soils without deficiency symptoms, legumes and crucifers show pronounced visual and physiological signs of Mo deprivation [9,135]. Table 7 shows the sensitivity categories for Mo in the different plants.

In the case of insufficient Mo supply, in addition to the above, sugar and chlorophyll contents decrease [58,121,140]. Furthermore, the intensity of photosynthesis and biosynthesis of ascorbic acid are inhibited [141,142]. In the leaf tissues of plants deficient in Mo, nitrate accumulates and the protein content of the plant decreases. Dicotyledonous plants are more susceptible to Mo deficiency than are monocots [143].

**Figure 14:**
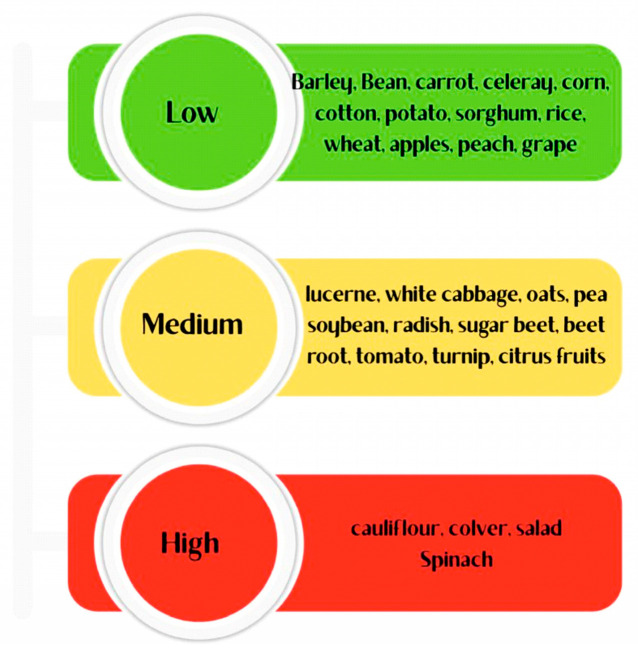
Categories of molybdenum sensitivity of different plants [50,111,134].

The need for Mo is high in cruciferous and leguminous plants. These crops often show significant Mo deficiency symptoms in soils where cereals can be grown without abnormalities associated with Mo deficiency. The cruciferous and leguminous plants listed in Figure 14 exhibit significant Mo-deficiency symptoms [50,117,144].

In dicotyledons, the following deficiency symptoms may occur with Mo deficiency: leaf distortions, wilted leaf plates, chlorotic lesions, necrotic spots, and necrotic necrosis [76,135]. Some plants are more sensitive to Mo deficiency; therefore, they can be used as indicator plants. Examples of such indicator crops include oilseed, peas, cauliflower, cabbage, tomatoes, lettuce, and spinach [42,69,136,137,138,139,140]. In cauliflowers, the leaf plate becomes increasingly pronounced as Mo deficiency intensifies; in extreme cases, it resembles a whiplash. These are symptoms of a deficiency disease called “whiptail” [58,141]. Another name for the disease is filamentous leafiness because in plants showing deficiency, the surface area of the leaf plate is greatly reduced, and on older leaves, only small torn leaf plate residues can be found along the main vein [142].

#### 1.3.8. Effect of Sufficient Molybdenum on Plant Development Across Various Soil Types

As mentioned above, Mo is the nutrient with the smallest, or consequently the smallest, amount after nickel and is necessary for the smooth functioning of plants [58]. However, numerous studies have shown that using a higher amount of Mo in addition to the needs of plants has a positive effect on both plant development and yield [44].

The addition of molybdenum to peas at the leaves and beginning of flowering increases the number of flowers and pods, as well as the yield and total protein content of green peas [81,143].

Gungula [144] used Mo doses (0–0.39 kg ha^−1^) in the cultivation of sand beans and investigated how different doses of molybdenum affect the yield of sand beans. It was found that crop yields were lowest in the control treatment (1069 kg ha^−1^), while 1280 kg ha^−1^ and 1250 kg ha^−1^ were harvested at 0.26 kg ha^−1^ dose and 1250 kg ha^−1^ at 0.39 kg ha^−1^.

According to a study conducted by [145], the Mo dose in loamy sand soil ensures an increase in chickpea yields. The highest yield was achieved by applying a dose of 80 g ha^−1^ of Mo in the form of ammonium molybdate, which increased the yield of stems by 5.75 m.m% and shoots by 9.54 m.m% (compared to plants that did not receive Mo). Ref. [146] investigated the supply of Mo to chickpeas through a field experiment. Mo concentration plays an important role in increasing crop yield. Based on their results, it was established that if a dose of 241 g ha^−1^ Mo was used for chickpea cultivation, an increase of more than 26% in the dry weight of pea pods, almost 20% in the dry mass of plants, and approximately 25% in the yield of peas could be achieved. Based on previous studies, Mo is more effective as a foliar fertilizer.

The quality and yield of sunflowers were investigated in field trials, during which the effects of Mo on the yields of sunflower crops were studied. Skarpa [147] found that the application of 125 g ha^−1^ foliar fertilizer resulted in a significant increase in sunflower biomass. Furthermore, Mo increased the oil yield of sunflowers to a small extent (compared to that of the control), albeit substantially.

Karpagam [148] conducted field experiments (on sandy clay soils) to investigate the effects of molybdenum treatment on the development and yield of mungo beans. Mo was administered as sodium molybdate at 0, 0.2, 0.4, 0.6, 0.8, and 1 kg ha^−1^. Based on their studies, a Mo dose of 1 kg ha^−1^ proved to be the most appropriate for both plant development and crop yield. In this treatment, approximately twice the root length, nearly three times the dry mass of the fruit, and an almost two-fold increase in the dry mass of the shoot were detected (compared to the control plants).

#### 1.3.9. Occurrence of Molybdenum Toxicity in Plants

Different plants react more visibly and sensitively to the phenomenon of Mo deficiency than to the effect of its high concentration, which causes Mo toxicity. In many cases, Mo can accumulate at such high concentrations in alkaline soils with high calcareous content and in floodplain areas, which can cause toxic effects; however, this also occurs only at very high concentrations [41,110]. For nitrogen metabolism in plants, a Mo content of 0.1–0.5 mg kg^−1^ is usually sufficient, but during the analysis of plants, it has often been found that Mo concentration of 10–50 mg kg^−1^ causes toxic symptoms even in the cultivation of several plant species. Many leguminous and cruciferous plants require relatively high Mo levels. The most obvious symptom of Mo toxicity is leaf chlorosis, which ranges from golden yellow to orange [58]. Other symptoms of Mo toxicity include inhibited elongation of taste spaces, growth of axillary buds, succulents of older leaves, and thickening of plant stems [41]. Additional toxicity symptoms attributable to excessive Mo concentrations can be considered, such as retarded development of green pea roots and shoots [149].

In terms of Mo accumulation, different plant types (monocotyledonous and dicotyledonous) showed significant differences. Brassica ssp. accumulated the highest amount of Mo in the vacuoles of epidermal cells (4762 mg kg^−1^) [131], and even sand beans (*Vigna unguliculata* L.) accumulated Mo at concentrations of 30.26 mg kg^−1^ [51]. However, in maize (*Zea mays* L.), the range is from 42.8 to 58.4 mg kg^−1^ [64].

In addition to the specific plant species, the actual molybdenum content of plants depends on the amount of molybdenum available to the plant in the soil, that is, the mobile molybdenum content in the soil. Individual plant species, especially leguminous plants, can accumulate large amounts of Mo in their bodies, which can reach up (<350 mg kg^−1^) without any toxic symptoms [111]. Moreover, the molybdenum concentration in cabbage was found to be 1061 mg kg^−1^ [39,150]. The leaves of many plant species have a higher Mo content than their stems. Examples include tomatoes, alfalfa, and soy [58].

Even with relatively high concentrations of molybdenum in plants, phytotoxic symptoms are rarely observed; however, high concentrations of molybdenum in plants pose a significant risk, as high concentrations of molybdenum in animal nutrition, especially in ruminants, can cause molybdenosis. The risk of developing the disease can occur even above 5 mg kg^−1^ Mo, which cannot be forgotten when feeding animals [58,110].

#### 1.3.10. Effects of Molybdenum on Macronutrient Uptake in Plants

Molybdenum treatment has a synergistic effect on certain elements (promoting their uptake) and an antagonistic effect (inhibiting their uptake) on other elements such as nitrogen, phosphorus, and sulfur [151]. As Mo is a crucial element for enzymes that play a significant role in nitrogen metabolism in plants, it naturally promotes nitrogen uptake [13,47,110] reported that applying 40 g ha^−1^ Mo to the surface of beans (*Phaseolus vulgaris* L.) in the form of foliar fertilizer increased the total nitrogen content of the grain crop by 10%. Fabio [152] also applied 78 g ha^−1^ Mo as a foliar fertilizer, resulting in a 142% increase in the total N content of sunflower leaves (compared to the controls). According to a previous study [147], the application of 125 g ha^−1^ Mo as a foliar fertilizer caused a significant increase in nitrogen content in all sunflower plant parts. In addition, ref. [153] Yu reported a positive correlation between molybdenum and nitrogen content in winter wheat barley [154].

The effect of Mo on phosphorus (P) uptake varies depending on the plant species, Mo and P supply levels, and interactions with other nutrients. In a study on the interaction of Mo with phosphorus (P), conflicting results were reported. For example, Liu [149] found that Mo (0, 0.01, 0.1, and 1 mg dm^−3^) used in their nutrient solution experiment increased phosphorus concentration in the roots and shoots of rapeseed (*Brassica napus*). In a nutrient solution experiment [155], increasing Mo treatments (0, 0.01, and 1 mg dm^−3^) significantly increased the phosphorus concentration in rice (*Oryza sativa*) shoots, but did not have a significant effect on the phosphorus concentration in the roots.

The interface between Mo and P in Brassica napus showed that P usage boosted shoot growth, while a low concentration of Mo supply (0.01 mg L^−1^) increased both shoot and root development. At higher P levels, Mo addition further improves P uptake in plant shoots and promotes the translocation of P from shoots to roots [149].

A study conducted on wheat (*Triticum aestivum* L.) grown in saline soil showed that Mo, P, and S significantly improved the grain yield and nutrient uptake. The highest yield (1.91 t/fed) was achieved with 200 kg S/fed, 22.5 kg P/fed, and 100 g Mo/fed. P and S improved leaf N, P, K, and S levels, whereas sulfur reduced leaf Mo, and both phosphorus and Mo increased Mo accumulation [156].

The plant Mo level increased due to the Mo supply across different plant species, such as *Leymus chinensis*, *Stipa baicalensis*, and *Carex duriuscula*, by enhancing soil Mo availability. In the absence of Mo addition, nitrogen reduced Mo uptake in *L. chinensis* (irrespective of mowing) and in *S. baicalensis* with mowing. When Mo was applied, nitrogen further repressed Mo absorption in all studied species, which was expected because of increased sulfur uptake antagonizing Mo and reduced phosphorus concentration, weakening (Mo–P interactions), except in *C. duriuscula*. Mowing reduced the Mo level only in *S. baicalensis* without nitrogen addition under Mo supply [157].

Hunashikatti [158] found that 1 kg ha^−1^ Mo increased sulfur accumulation by approximately 25% in the leaf and stem parts, and by 20% in the head of cabbage, whereas in the highest treatment (2 kg ha^−1^), a decrease was found in all parts of the cabbage (30–35%). Rabbi [159] reported that Mo treatment of 0.4 and 0.8 kg ha^−1^ applied in field pea experiments significantly increased the sulfur content of plant samples (compared to the control crop).

#### 1.3.11. Effects of Molybdenum on Micronutrient Uptake by Plants

**Figure 15:**
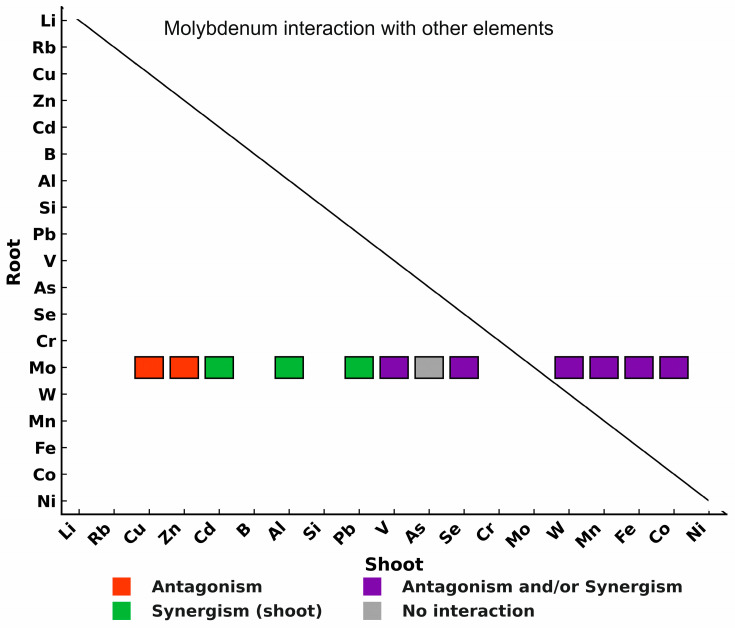
Major synergistic and antagonistic interactions between Mo and microelements in plants. Mo generally exhibits antagonism with Cu, Fe, and Mn, with the Mo-Cu antagonism closely linked to nitrogen metabolism. Mo-Mn interactions are antagonistic in roots but can show both synergistic and antagonistic effects in shoots, influenced by soil conditions. Synergistic interactions are observed with boron (B) and silicon (Si), while Mo may also affect W and V uptake due to biochemical replacement. High Mo levels can induce Fe deficiency, likely through Fe-Mo precipitation in roots.

Of course, molybdenum affects the uptake of not only macroelements but also many microelements. The synergistic and antagonistic relationships between Mo and the most notable microelements are shown in Figure 15.

Several complex interactions can be observed between Mo and other elements in the plant tissues and roots. Figure 15 shows that Mo influenced the uptake of B, Cu, Fe, Mn, Si, and W. There is antagonism between Mo and the former elements, except for boron and silicon, which is also reflected in the elemental content of the roots and shoots of the plant. The most important interaction between Mo and the microelements is copper. Mo-Cu antagonism in plants is closely related to N metabolism [150]. As shown in Figure 15, a clear antagonistic relationship between Mo and Mn was established only in the roots; however, a synergistic and antagonistic relationship between Mo and Mn may exist in the shoots. Because Mo-Mn antagonism due to soil acidity affects the concentrations of these elements (Mo and Mn) available to plants, liming can correct for both Mo deficiency and Mn toxicity. The Mo-Fe interaction can be best illustrated by the low concentration of Mo in the solution of iron-rich soils. However, a very high Mo content can also induce iron deficiency. Although the mechanisms of interactions within plants are not sufficiently understood, Fe-Mo precipitation at the roots is likely responsible for the lack of sufficient Mo translocation. In addition, Mo promotes the uptake of Si (synergism) in the shoots of plants; therefore, the addition of Mo increased the concentration of Si in the shoots of plants. Metabolic interactions between Mo, W, and Mo-V cannot be excluded because these elements are highly likely to replace Mo in many biochemical processes.

It can also be observed from the data in Figure 15 that the management of Mo does not have a synergistic or antagonistic effect on the uptake of Se as an anion with a similar structure. This fact is supported by the publication of [60], in which strawberries were examined using 0–202.5 g ha^−1^ Mo treatment. Ref. [160] investigated the effect of Mo treatment on rice (Oryza sativa) micronutrient uptake under nutrient solution (hydroponic) conditions. Mo and ammonium molybdate [(NH4)_6_Mo_7_O_24_⋅4H_2_O] were added to the medium at four different concentrations (0, 0.01, 0.1, and 1 mg dm^−3^), and five different rice varieties were used for the studies. Lu et al. [161] conducted experiments with rice test plants and found that Mo treatment significantly increased the concentration of Mn in rice sprouts but did not have a significant effect on the Mn content of the root. Nadia [162] reported that in a field experiment, the application of 12 mg kg^−1^ Mo significantly increased the concentration of Cu in peanut crops compared with that in control crops. They also investigated the effect of 16 mg kg^−1^ Mo application in sand bean grains and found that Mo promoted the uptake of all the examined microelements (Mn, Fe, Cu, and Zn), including copper.

## 2. Conclusions

This review integrates bibliometric mapping with physiological and agronomic analyses to evaluate the effects of molybdenum application on plant growth and crop production. The research centers on two main themes: the biochemical roles of molybdenum and its agronomic impact on soil fertility and crop yield. Since 2016, the focus has shifted from molecular studies to applied, field-based research emphasizing sustainable agriculture and nutrient-use efficiency. Despite progress, gaps remain in large-scale field validation, crop- and soil-specific Mo requirements and applications, nutrient interactions under stress resistance, and molecular mechanisms related to signal transduction. Future studies should focus on multi-location trials, nutrient interaction experiments, and crop-specific Mo management using advanced omics and agronomic methods. The limitations include the reliance on English, open-access Web of Science publications and the variability across studies, although the combined approach offers a solid foundation for further research on this topic, which is linked to the Sustainable Development Goals.

## Figures and Tables

**Table 1:** The top 20 corresponding authors’ countries of (MoAPG) and crop production documents (1999–2025), based on the WoS database.

Country	Articles	Articles %	SCP	MCP	MCP %
**CHINA**	80	16	56	24	30
**BRAZIL**	67	13.4	57	10	14.9
**USA**	53	10.6	38	15	28.3
**INDIA**	39	7.8	37	2	5.1
**CANADA**	20	4	13	7	35
**AUSTRALIA**	19	3.8	14	5	26.3
**POLAND**	16	3.2	14	2	12.5
**ITALY**	15	3	9	6	40
**IRAN**	14	2.8	11	3	21.4
**JAPAN**	13	2.6	10	3	23.1
**SPAIN**	13	2.6	10	3	23.1
**UNITED KINGDOM**	12	2.4	4	8	66.7
**GERMANY**	10	2	7	3	30
**ISRAEL**	9	1.8	3	6	66.7
**SOUTH AFRICA**	8	1.6	8	0	0
**CZECH REPUBLIC**	6	1.2	6	0	0
**MEXICO**	6	1.2	6	0	0
**BANGLADESH**	5	1	3	2	40
**FRANCE**	5	1	4	1	20
**KOREA**	5	1	1	4	80

**Table 2:** The top 20 most cited countries on (MoAPG) and crop production documents (1999–2025) based on WoS.

Country	Total Citation	Average Article Citations
**JAPAN**	2059	158.40
**CHINA**	1877	23.50
**USA**	1778	33.50
**UNITED KINGDOM**	1193	99.40
**BRAZIL**	1100	16.40
**GERMANY**	453	45.30
**ITALY**	353	23.50
**ISRAEL**	343	38.10
**CANADA**	307	15.30
**SPAIN**	284	21.80
**INDIA**	224	5.70
**AUSTRALIA**	219	11.50
**IRAN**	203	14.50
**POLAND**	199	12.40
**SOUTH AFRICA**	166	20.80
**FRANCE**	141	28.20
**KOREA**	129	25.80
**PORTUGAL**	112	37.30
**BELGIUM**	108	36.00
**PHILIPPINES**	105	105.00

**Table 3:** Molybdenum content in different plants [47].

Plant Sample	Concentration (mg kg^−1^)
Wheat (grain)	0.2–2.4
Spring barley (grain)	0.17–1.2
Rye (grain)	0.12–1.3
Oat (grain)	0.28–1.9
Peas (seeds)	1.2–1.8
Seed beans (seeds)	0.9–1.6
Carrot (root)	0.04
Onion (head)	0.16–0.24
Potatoes (tubers)	0.1–0.25
Tomato (berry)	0.82
Apples (fruit)	0.07

**Table 4:** Molybdenum content of different plant leaves in the dry matter (mg kg^−1^) [41].

Plant	Mo Concentration (mg kg^−1^)	
	Lack	Need
Sugar beet (*Beta vulgaris* L.)	˂0.16	0.2–20
Maize (*Zea mays* L.)	˂0.12	1.4–7.0
Alfalfa (*Medicago sativa* L.)	˂0.4	0.5–5.0
Beans (*Phaseolus vulgaris* L.)	˂0.2	0.2–5.0
Cabbage (*Brassica oleracea* L.)	˂0.3	0.3–3.0
Cucumbers (*Cucumis sativus* L.)	˂0.2	0.2–2.0
Red clover (*Trifolium pratense* L.)	˂0.15	0.3–1.59
Peas (*Pisum sativum* L.)	-	0.4–1
Barley (*Hordeum vulgare* L.)	-	0.09–0.8
Sunflower (*Helianthus annuus* L.)	-	0.25–0.75
Oatmeal (*Avena sativa* L.)	-	0.2–0.3
Lettuce (*Lactuca sativa* L.)	˂0.07	0.08–0.14

**Table 5:** Molybdenum concentration in plants as a function of plant age (mg kg^−1^) [41].

Plant Species	30 April	12 May	26 May	16 June
Lucerne	0.624	0.520	0.279	0.177
Meadow red clover	1.149	0.416	0.520	0.377
Field red clover	1.308	1.281	1.094	0.886
Meadow fescue	0.485	0.614	0.692	0.282
Rye	0.398	0.263	0.278	0.261
Wheat	0.378	0.324	0.386	0.118

**Table 6:** Molybdenum application effects, sufficient amounts, and outcomes across different plants/crops.

Plant/Crop	Plant Part/Context	Mo Metric	Value	Outcome/Notes	Citation
Sugar beet (*Beta vulgaris* L. ssp. *vulgar*)	Leaf plates	Tissue Mo concentration	<0.16 Deficiency symptom	Reference sufficiency ranges from 0.2 to 20.0	[13]
Millet variety of (Changnong 47)	Foliar Mo at jointing with N = 75 kg/ha	Foliar Mo concentration	0.3% (Mo_3_)	Max yield 5869 kg/hm^2^ (+13% vs. no fertilization); total dry matter 36.96 g/plant (+31%).	[111]
Aromatic rice Xiangyaxiangzh under Cd stress	Soil application; pot experiment with Cd 100 mg/kg	Soil-applied Mo rate	0.15 mg Mo/kg soil	Grain yield +64.75%; 2-acetyl-1-pyrroline +77.09%.	[112]
Peanut (Runneriac 886)	Seed inoculation with Bradyrhizobium and/or Azospirillum; Mo on seed	Mo on seed	200 mg Mo/kg seed	Root nodulation, dry matter, chlorophyll ↑; grain yield +17.7% (2017–2018).	[113]
Peanut (cv. IAC Tatu ST)	Bradyrhizobium inoculation + foliar Mo on sandy soil	Foliar Mo rate (optimal range)	80–100 g Mo/ha	Grain yield +24% in 2016/2017; nodulation, leaf N, and shoot dry matter.	[114]
Soybean in maize–soybean intercropping	Field, sodium molybdate applied at four levels	Soil/foliar Mo rate (as Na_2_MoO_4_)	120 g Mo/ha	Highest growth and yield; intercropped grain yield 626 and 725 kg/ha; +15% and +20% vs. control (2020/2021).	[67]
Wheat (sandplain, SW Australia)	Naturally acidic soils; seed Mo history	Critical YEB Mo concentration; fertilizer rate to achieve 90% max yield	0.08–0.09 mg Mo/kg (YEB); 55 g/ha (low-Mo seed) vs. 15 g/ha (high-Mo seed)	Higher Mo in sowing seed reduces fertilizer Mo needed; grain yield response 59% (low-Mo seed) vs. 15% (high-Mo seed).	[115]
Common bean (Phaseolus vulgaris)	Two Oxisols; Rhizobium inoculation ± foliar Mo	Foliar Mo rate	80 g Mo/ha	Yield response depended on soil chemistry; the highest yield at Patos de Minas site.	[116]

**Table 7:** Molybdenum sensitivity categories of different plants.

Mo Sensitivity	Plant Examples	References
High	- **Crucifers**: Cauliflower, cabbage, turnip, radish, spinach - **Legumes**: Lucerne (alfalfa), clover, pea, soybean, bean, chickpea, lentil	[8,51,56,136]
Medium	Oats, beetroot, tomato, sugar beet	[137,138,139]
Low	Barley, wheat, rice, sorghum, corn (cereals); carrot, potato; apple, citrus, grape, peach	[117,134]

## Data Availability

No new data were created or analyzed in this study. Data sharing is not applicable to this article.
